# Microrna 130b Suppresses Migration and Invasion of Colorectal Cancer Cells through Downregulation of Integrin β1

**DOI:** 10.1371/journal.pone.0087938

**Published:** 2014-02-03

**Authors:** Yanyang Zhao, Gang Miao, Yao Li, Tomoya Isaji, Jianguo Gu, Jian Li, Ruomei Qi

**Affiliations:** 1 The Key Laboratory of Geriatrics, Beijing Hospital & Beijing Institute of Geriatrics, Ministry of Health, Beijing, China; 2 Department of Surgery, Beijing Hospital, Ministry of Health, Beijing, China; 3 Division of Regulatory Glycobiology, Institute of Molecular Biomembrane and Glycobiology, Tohoku Pharmaceutical University, Sendai, Miyagi, Japan; The University of Hong Kong, China

## Abstract

MicroRNA 130b (miR-130b) is significantly dysregulated in various human tumor types. In this study, using a microarray assay, we characterized the upregulation of miR-130b expression in colorectal cancer (CRC) specimens. However, there is limited knowledge about the roles of aberrant miR-130b expression in CRC. Our studies in CRC cells demonstrated that miR-130b significantly decreases cell migration and invasion, but it has no evidently effects on cell proliferation and apoptosis. In the overexpression miR-130b CRC cells and the CRC specimens, we observed a decreased level of integrin β1 protein, which is considered as a key molecule involved in cell motility. The targeting of the 3′-UTR region of integrin β1 gene by miR-130b was revealed using a luciferase reporter assay. The regulation of integrin β1 by miR-130b was further shown using the miR-130b mimics and the inhibitor of miR-130b. The impaired motility of the miR-130b overexpression cells is recovered partly by the expression of integrin β1 lacking the 3′-UTR. Additionally, the knockdown of integrin β1 also gives rise to a decrease in cell migration and invasion, which is similar to the impeded motility due to overexpression of miR-130b in CRC cells. Furthermore, the inverse expressions of miR-130b and integrin β1 were observed in CRC specimens. In summary, these data demonstrate that miR-130b downregulates its target-integrin β1, leading to the impaired migration and invasion of CRC cells.

## Introduction

MicroRNAs (miRNA) are short non-coding RNAs of 24 to 25 nucleotides that mediate gene silencing through imperfect hybridization to 3′ untranslated region (3′-UTR) in target mRNAs [1]. MiRNAs play important roles in virtually all biological activities in mammals and other multicellular organisms [2]. Moreover, it has been reported that miRNAs influence numerous cancer-relevant processes such as migration, proliferation. More importantly, microRNA molecules are already entering the clinic as diagnostic and prognostic biomarkers for patient stratification and also as therapeutic targets and agents [3]. Recently, miR-130b is revealed as one of novel tumor-related miRNAs and has significantly dysregulated in tumors by a comprehensive meta-analysis of miRNA expression microarray datasets, which comprises 33 comparisons and nearly 4,000 tumor and corresponding nontumors samples [4]. Accordingly, miR-130b has been found upregulated in various types of cancer: gastric cancer [5], [6], cutaneous malignant melanoma [7], head and neck squamous cell carcinoma [8] and bladder cancer [9]. Together, it has been estimated that miR-130b plays key roles during oncogenesis.

Colorectal cancer (CRC) is the third most commonly diagnosed cancer in men and the second in women worldwide. Approximately 608,000 deaths from colorectal cancer are estimated worldwide, making it the fourth leading cause of cancer death [10]. Currently, one of the obstacles in cancer treatment is the high rate of tumor metastasis. The metastatic process follows of a series of steps: first, cancer cells within the primary tumor break away from neighboring cells and invade the basement membrane. This local invasion may frequently be triggered by contextual signals that causing cancer cells to undergo an epithelial-mesenchymal transition (EMT) [11]. After intravasation, the cells might extravasate from the circulation into the surrounding tissue, where they may remain dormant or initiate and maintain growth to form angiogenic metastases [12], [13]. Metastasis is the major cause of death in many cancers, including CRC [14]–[16]. Therefore, a better understanding of the molecular mechanisms underlying metastasis is required to facilitate the development of effective therapeutic strategies for patients with CRC.

In our study, we compared miRNA expression in specimens from CRC patients using a microRNA microarray and observed the significant upregulation of miR-130b expressed in the CRC specimens. To gain insight on the roles of miR-130b in CRC, we investigated the effects of miR-130b in CRC cells and CRC specimens. Our data suggested that integrin β1 is a target gene of miR-130b and the downregulation of integrin β1 by miR-130b leads to the impaired migration and invasion of CRC cells.

## Experimental Procedures

### Clinical specimens

Colorectal cancer and adjacent control tissue specimens were obtained from 33 patients at Beijing Hospital, Ministry of Health (Beijing, China) after surgical resection. The tumor tissues and adjacent normal tissues were frozen in liquid nitrogen after resection. No patient in the current study received chemotherapy or radiation therapy before the surgery. All patients provided written informed consent for the use of their tissues, according to the Declaration of Helsinki. The study protocol was approved by the Ethics Committee of Beijing Institute of Geriatrics, Ministry of Health.

### MicroRNA microarray analysis

Small RNAs were isolated from tumor tissues and adjacent normal tissues. The quality control, labeling, hybridization and scanning procedures were performed by CapitalBio (Beijing, China), using the Affymetrix's GeneChip miRNA array chip V1.0. Differentially expressed genes between tumor tissues and adjacent normal tissues were analyzed using the SAM software 3.02. MiRNAs that fulfilled the criteria of q value (%)≤5 and fold change ≥2 or fold change ≤0.05 between groups were considered to be significantly different. Heat map was performed using Cluster 3.0 package software. The data presented in this study have been deposited in the National Center for Biotechnology Information (NCBI) Gene Expression Omnibus (GEO) and are accessible through the GEO accession number GSE53592.

### Cell culture

The human colorectal cancer cell lines SW-480 and SW-620 were purchased from the Cell Resource Center, IBMS, CAMS/PUMS and passed in less than 6 months. Cells were cultured in RPMI-1640 (Gibco, Paisley, UK) with 10% FBS (Gibco, Paisley, UK) and 2 mmol/L L-glutamine (Gibco, Paisley, UK), 100 U/ml of penicillin (Gibco, Paisley, UK), and 100 µg/ml of streptomycin sulfate (Gibco, Paisley, UK).

### Pri-miR-130b cloning, lentivirus production and transduction

The human primary microRNA 130b gene (pri-miR-130b) was amplified by PCR from human genomic DNA using the following primers: Forward 5′-ATATTCTCGAGGGGGATCTCCC-3′ and Reverse 5′-ATATCGGATCCTCTTACCCCAG-3′, and then subcloned into the pLVX-IRES-Hyg vector (TaKaRa, Dalian, China) to generate pLVX-miR-130b. The virus particles were harvested 48 h after the transfection of pLVX-miR-130b into HEK-293T cells using the Lenti-HT packaging mix (TaKaRa, Dalian, China). The SW480 cells were infected with the harvested recombinant lentivirus in the presence of 6 µg/ml Polybrene (Sigma, St Louis, USA). The SW480 cells were maintained in complete growth medium in the presence of 1 mg/ml Hygromycin (Roche Applied Science, Mannheim, Germany). The PCR amplicon of pri-miR-130b was also subcloned into the pWPI lentiviral vector (Addgene, Cambridge, MA, USA) to generate pWPI-miR-130b. The empty pWPI vector, encoding green fluorescent protein (GFP), was used as the control. The virus particles were harvested as described earlier. The SW620 cells stably expressing GFP were selected by fluorescent-activated cell-sorting (FACS), with the use of a Vantage SE Diva cell sorter (Becton Dickinson, Franklin Lakes, NJ, USA).

### RNA reversed transcription and quantitative real-time PCR (qRT-PCR) assays

The total RNA, including small RNAs, was extracted from the clinical specimens or from the CRC cells with a miRVana MicroRNA Isolation Kit (Invitrogen, Carlsbad, USA) and subjected to reverse transcription. qRT-PCR was performed using with the SYBR Premix Ex Taq mix (TaKaRa, Dalian, China) according to the manufacturer's instructions, and the samples were run on an iQ5 Multicolor Real-time PCR Detection System (Bio-Rad, Hercules, USA). Thermal reaction cycles of 95°C for 30 s, and 45 repetitions of 95°C for 5 s and 60°C for 20 s were used. The primers used were as follows: hsa-miR-130b Forward 5′-GCCGCCAGTGCAATGATGAA-3′ hsa-miR-130b Reverse 5′-GTGCAGGGTCCGAGGT-3′; U6 Forward 5′-CGCTTCGGCAGCACATATACTA-3′; U6 Reverse 5′-CGCTTCACGAATTTGCGTGTCA-3′

### Luciferase reporter assay

The full-length 3′-untranslated region (3′-UTR) fragments of the integrin β1 gene were amplified by PCR from human genomic DNA using primers Forward 5′-GTACTGCCCGTGCAAATCCCACAAC-3′ and Reverse 5′-TGCTTTTCCTCAACTTCTTTAATC-3, and were cloned into a pMD18-T vector (TaKaRa, Dalian, China). The *Sac*I-*Sal*I-digested products were cloned into a pmirGlo Dual-luciferase miRNA Target Expression Vector (Promega, Madison, USA) to form 3′-UTR-luciferase reporter vector. The SW480 cells were cotransfected in 24-well plates using Lipofectamine 2000 (Invitrogen, Carlsbad, USA) with 3′-UTR-luciferase reporter vector and the indicated miRNAs. Twenty-four hours after transfection, firefly and Renilla luciferase activities were measured consecutively using dual-luciferase assay (Promega, Madison, USA), according to the manufacturer's protocols. Negative control vector were generated by cloning the same 3′-UTR of integrin β1 gene in reverse orientation. The activity of samples was measured in a GloMax 20/20 Luminometer (Promega, Madison, USA). The firefly luciferase activity was normalized by Renilla luciferase activity for transfection efficiency.

### Cell transfection

The plasmid used for the expression of integrin β1 lacking the 3′-UTR (β1-ORF) was described previously [17]. We used pWPI vector as a control. The hsa-miR-130b mimics, hsa-miR-130b inhibitor (anti-miR-130b), control mimics and siRNA against integrin β1 [18] were synthesized by Ribobio (Guangzhou, China). The sequences used were as follows: hsa-miR-130b mimics, 5′-CAGUGCAAUGAUGAAAGGGCAU-3′; hsa-miR-130b inhibitor, 5′-AUGCCCUUUCAUCAUUGCACUG-3′; integrin β1 siRNA, (sense) 5′-GGAACAGCAGAGAAGCUCA-3′ [18]; The SW480 cells were transfected using RNAiMax (Invitrogen, Carlsbad, USA) or Lipofectamine 2000 (Invitrogen, Carlsbad, USA) according to the manufacturer's instructions.

### Cell migration assay and invasion assay

Cell migration assay was evaluated using transwell chambers (8-µm, BD Bioscience,San Jose, USA). 5×10^5^ cells were placed into the upper chamber of each insert, and 500 µl of complete medium was added to the bottom well. The cells that not migrated were removed from the upper surfaces of the filters using cotton swabs, and the cells that had migrated to the lower surfaces of the filters were fixed with 4% paraformaldehyde solution and stained with 0.1% crystal violet. Images of three random fields were captured from each membrane, and the number of migratory cells was counted. Similar inserts coated with matrigel were used to determine the invasive potential.

### Cell proliferation assay

Cell proliferation was determined using a tetrazolium compound [3-(4,5-dimethylthiazol-2-yl)-5-(3-carboxymethoxyphenyl)-2-(4-sulfophenyl)-2H-tetrazolium, inner salt (MTS) (CellTiter 96 AQueous Non-Radioactive Cell Proliferation Assay) (Promega, Madison, USA), according to the manufacturer's protocol. Briefly, at the indicated times, assays are performed by adding the CellTiter 96 AQueous One Solution Reagent directly to culture wells, incubating for 2 h and then recording the absorbance at 490 nm with a 96-well plate reader.

### Western blotting

Proteins were separated by SDS-PAGE and transferred to a PVDF membrane (Millipore, Billerica, USA). The membrane was blocked with 5% non-fat milk and incubated with mouse anti-integrin β1 (1/2500, BD Biosciences, San Jose, USA), mouse anti-E-cadherin (1/2000, BD Biosciences, San Jose, USA), rabbit anti-caspase 3 (1/1000, Santa Cruz Biotechnology, Santa Cruz, USA), rabbit anti-caspase 8 (1/800, Millipore, Billerica, USA) or mouse anti-GAPDH (1/10000, Sigma, St Louis, USA) antibodies.

### Statistical analysis

The data are presented as the mean±s.d. The statistical significance between the groups was assessed by Student's *t*-test. A value of *P*<0.05 was considered as statistical significance.

## Results

### Detection of increased miRNA-130b levels in CRC specimens

**Figure 1:**
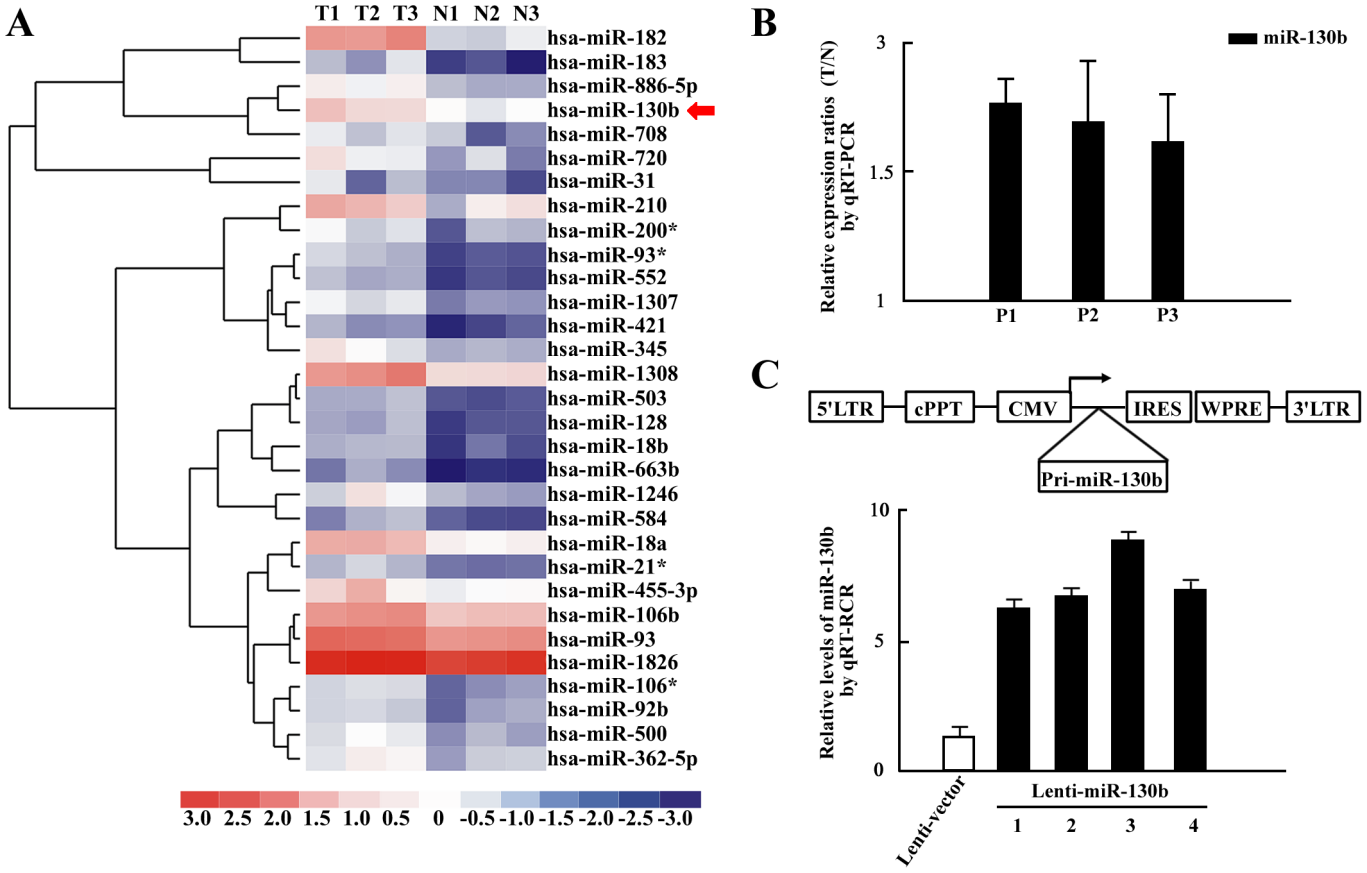
Increased miR-130b expression observed in CRC specimens. *A*, Heat map diagram of the 31 significantly elevated miRNAs in colorectal cancer tissues compared to matched adjacent control tissues from 3 patients (P1, P2, and P3). The adjacent control tissues are referred to N1, N2 and N3. The tumors are referred to T1, T2 and T3. MiRNAs are shown in rows. Samples are shown in columns. Red in the color bar indicates higher expression and blue indicates lower expression. MiR-130b (*Red Arrow*) is one of significantly upregulated miRNAs. *B*, Relative expressions of miR-130b (normalized to U6) in tumors over adjacent control tissues (T/N) from P1, P2, and P3, determined by qRT-PCR (mean±s.d.; n = 3). *C*, *Upper panel*: Schematic diagram of a lentiviral pLVX-IRES-Hyg vector containing the primary microRNA 130b (Lenti-miR-130b). *Lower panel*: Relative expressions of miR-130b (normalized to U6) in SW480 cells that stably expresses miR-130b (Lenti-miR-130b) or control (Lenti-control), examined by qRT-PCR (mean±s.d.; n = 3). The four colonies of Lenti-miR-130b cells are referred to 1,2,3,4.

Using a microarray assay, we characterized miRNA expression in colorectal cancer (CRC) tissues and marched adjacent normal tissues harvested from 3 patients (P1, P2, and P3). The characterization of the CRC patients is described in Table 1. We tested differential miRNA expressing using the SAM package. The 31 significantly upregulated miRNAs (Fig. 1A) probe sets (fold≥2) were identified in the CRC specimens. And the 17 significantly downregulated miRNAs were shown in Table S1. In the microarray readouts, we noticed that miR-130b is one of significantly upregulated miRNAs. A growing number of studies have reported miR-130b as tumor-related miRNA and miR-130b plays an important role during oncogenesis [4]. To better understand its potential functions in CRC, we firstly used qRT-PCR to confirm the miR-130b expression in the 3 CRC patients (Fig. 1B). Consistent with the microarray readouts in Fig. 1A, the qRT-PCR results revealed the enhanced expression of miR-130b in the 3 CRC tumor tissues (Fig. 1B). We then generated lentiviral vector expressing primary microRNA 130b (Lenti-pri-miR-130b) (Fig. 1C upper panel), and constructed SW-480 cells with stable overexpression of miR-130b (Lenti-miR-130b cells) and the respective control (Lenti-vector cells). We found that the levels of the mature miR-130b are significantly increased in the four colonies of Lenti-miR-130b cells, compared with Lenti-vector cells (Fig. 1C lower panel).

**Table 1:** Characterization of colorectal cancer patients used for microRNA microarray analysis.

Category	Characterization of colorectal cancer patients		
Patient	1	2	3
Sex	M	M	M
Age	52	92	31
Tumor size (cm^3^)	4.5×4.5×1−1.5	4×3×1.5	0.5−1.2×0.8×0.5
Regional lymphnode metastasis	0/27	4/6	1/7
Distant metastasis	No	No	No

### MiR-130b decreases migration and invasion of CRC cells

**Figure 2:**
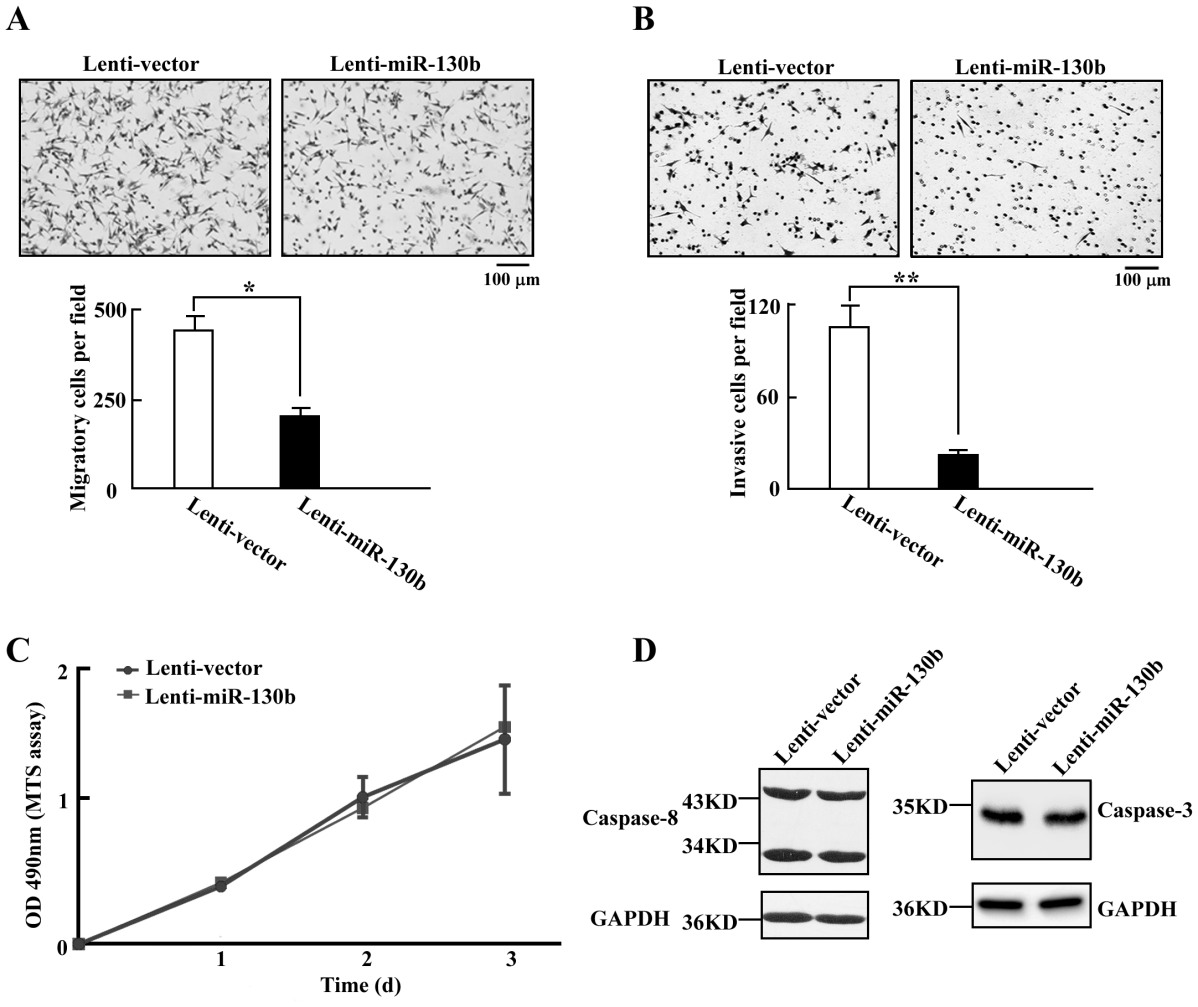
MiR-130b suppresses cell migration and invasion of CRC cells. *A,B*, Transwell migration (*A*) and invasion (*B*) assays of Lenti-control cells and Lenti-miR-130b cells (scale bar = 100 µm; mean±s.d.; n = 4; *, *P*<0.05; **, *P*<0.01). Representative images of migrated cells (*A*) and invaded cells (*B*) are shown. *C*, The proliferation of Lenti-control cells and Lenti-miR-130b cells measured by MTS assay during three-day time course (mean±s.d.; n = 4). *D*, Western blot analyses of caspase-3 and caspase-8 expression in Lenti-control cells and Lenti-miR-130b cells from three independent experiments. GAPDH used as loading control.

We next examined how miR-130b might function inside colorectal cancer cells. The Lenti-vector cells and Lenti-miR-130b cells were utilized to analyze the effects of miR-130b on CRC cells. The cells were firstly subjected to migration assay and invasion assay, respectively. We observed that miR-130b significantly inhibits the migration of the Lenti-miR-130b cells (Fig. 2A). We then examined the effect of miR-130b on the invasiveness of the cells using the matrigel invasion assay system. Consistent with the result of the migration assay, the invasiveness is significantly reduced in the Lenti-miR-130b cells (Fig. 2B). Interesting, there is no significant difference in proliferation between Lenti-vector cells and Lenti-miR-130b cells (Fig. 2C). Moreover, we didn't observe the obvious different expression levels of apoptotic caspases- caspase 3 and caspase 8 between Lenti-vector cells and Lenti-miR-130b cells (Fig. 2D). All these results suggested that overexpression of miR-130b results in the impaired cell motility of CRC cells, but it has no effect on proliferation and apoptosis of CRC cells.

### MiR-130b suppresses integrin β1 expression via its 3′-UTR

**Figure 3:**
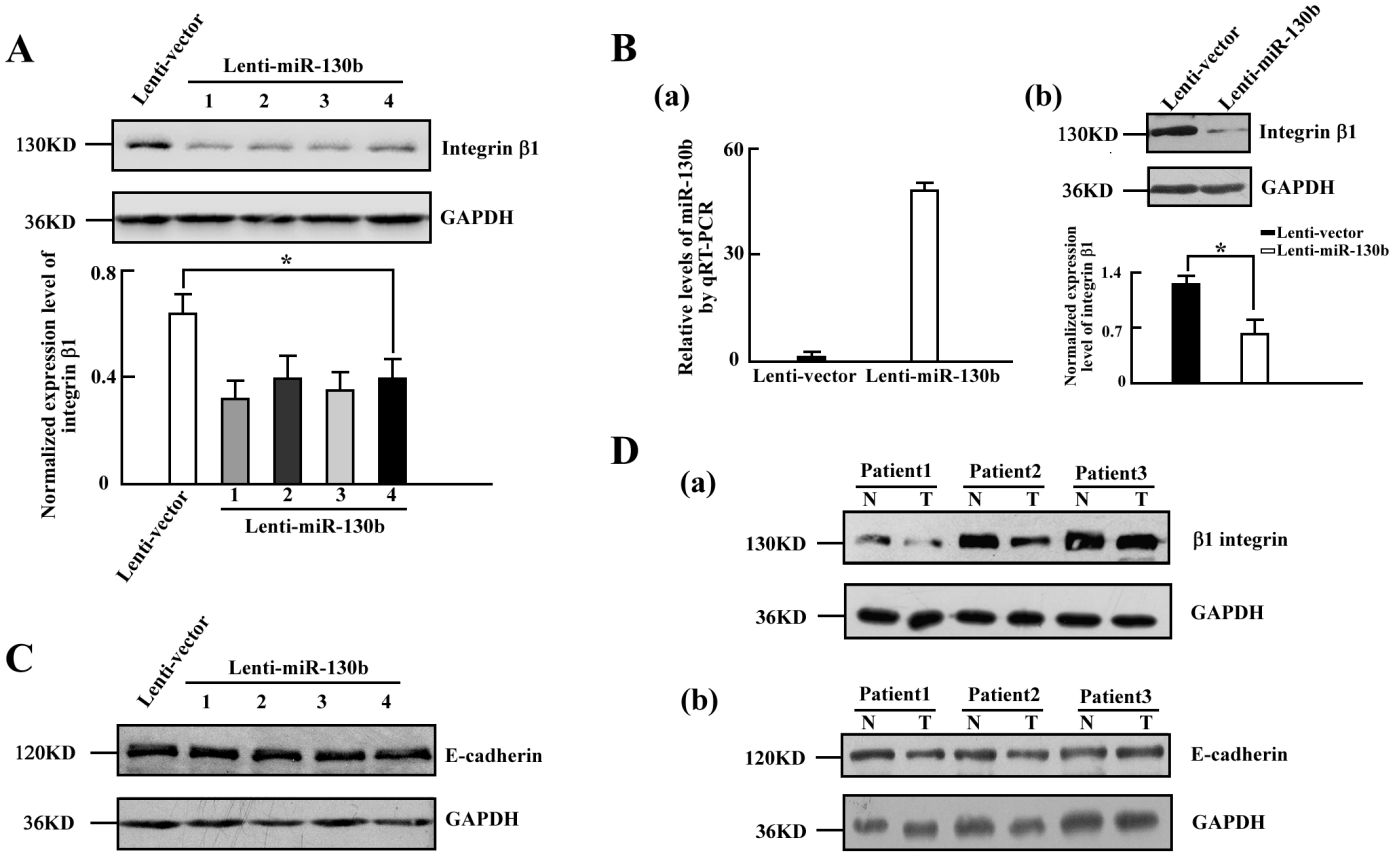
Elevated miR-130b results in decreased integrin β1 expression level. *A*, *Upper panel*: Western blot analyses of integrin β1 protein expression in Lenti-control cells and Lenti-miR-130b cells (SW480 cells) from three independent experiments. *Lower panel*: Densitometry analysis of the Western blot data normalized with GAPDH (mean±s.d.; n = 3; *, *P*<0.05). *B* (*a*), Relative expressions of miR-130b (normalized to U6) in SW620 cells infected with lenti-control virus or lenti-miR-130b virus, examined by qRT-PCR (mean±s.d.; n = 3). (*b*), *Upper panel*: Western blot analyses of integrin β1 expression in Lenti-control and Lenti-miR-130b cells (SW620 cells) from three independent experiments. *Lower panel*: Densitometry analysis of the Western blot data normalized with GAPDH (mean±s.d.; n = 3; *, *P*<0.05). *C*, Protein expression of E-cadherin analyzed by immunoblot in Lenti-control cells and Lenti-miR-130b cells from three independent experiments. The four colonies of Lenti-miR-130b cells (SW480 cells) are referred to 1,2,3,4. GAPDH used as loading control. *D*, The expression levels of integrin β1 (*a*) and E-cadherin (*b*) were determined by Western blot analyses using the matched the adjacent control tissues (N) the tumors (T) from the 3 colorectal cancer patients (Patient 1, Patient 2, and Patient 3). Each assay was independently repeated three times. GAPDH used as loading control.

We further investigated the mechanism by which miR-130b affects the motility of CRC cells. Our previous studies had shown that the post-translational modification plays an important role in integrin-mediated migration [17], [19], [20]. Integrins are a family of cell adhesion molecules comprising 18α and 8β subunits that combine into at least 24 heterodimers. More importantly, the cytoplasmic domain of integrin β1 transduces bidirectional signals from inside the cell by regulating the conformation and ligand affinities of the extracellular domain (inside-out signaling), while mediating downstream signaling and interactions with the cytoskeleton (outside-in signaling) [21]–[23]. So, integrin β1 is a key regulator involved in metastasis *in vitro* and *in vivo* [24]–[27]. In this study, we found that ectopic expression of miR-130b in SW480 cells results in a decrease in the endogenous integrin β1 protein level of four Lenti-miR-130b colonies by approximate 50%, compared with that of the Lenti-vector cells (Fig. 3A). The consistent result was observed in SW-620 cells with overexpression of miR-130b (Fig. 3B). However, there is no change in the expression level of E-cadherin (Fig. 3C), which is a key molecular involved in EMT. And as mentioned before, EMT is the initial step in metastasis. We also examined the expression of integrin β1 in the 3 pairs of specimens subjected to the microarray shown in Fig. 1A. Consistent with the data in Fig. 3A and Fig. 3C, the integrin β1 protein expression is decreased in the tumor tissues compared with the corresponding adjacent normal tissues (Fig. 3D (a)), whereas no obvious change of the expression of E-cadherin was detected (Fig. 3D (b)). It is notable that the decreased level of integrin β1 protein was detected in the overexpression miR-130b CRC cells (Lenti-miR-130b cells) and CRC specimens as well. Therefore, we sought to investigate whether miR-130b can regulate integrin β1.

**Figure 4:**
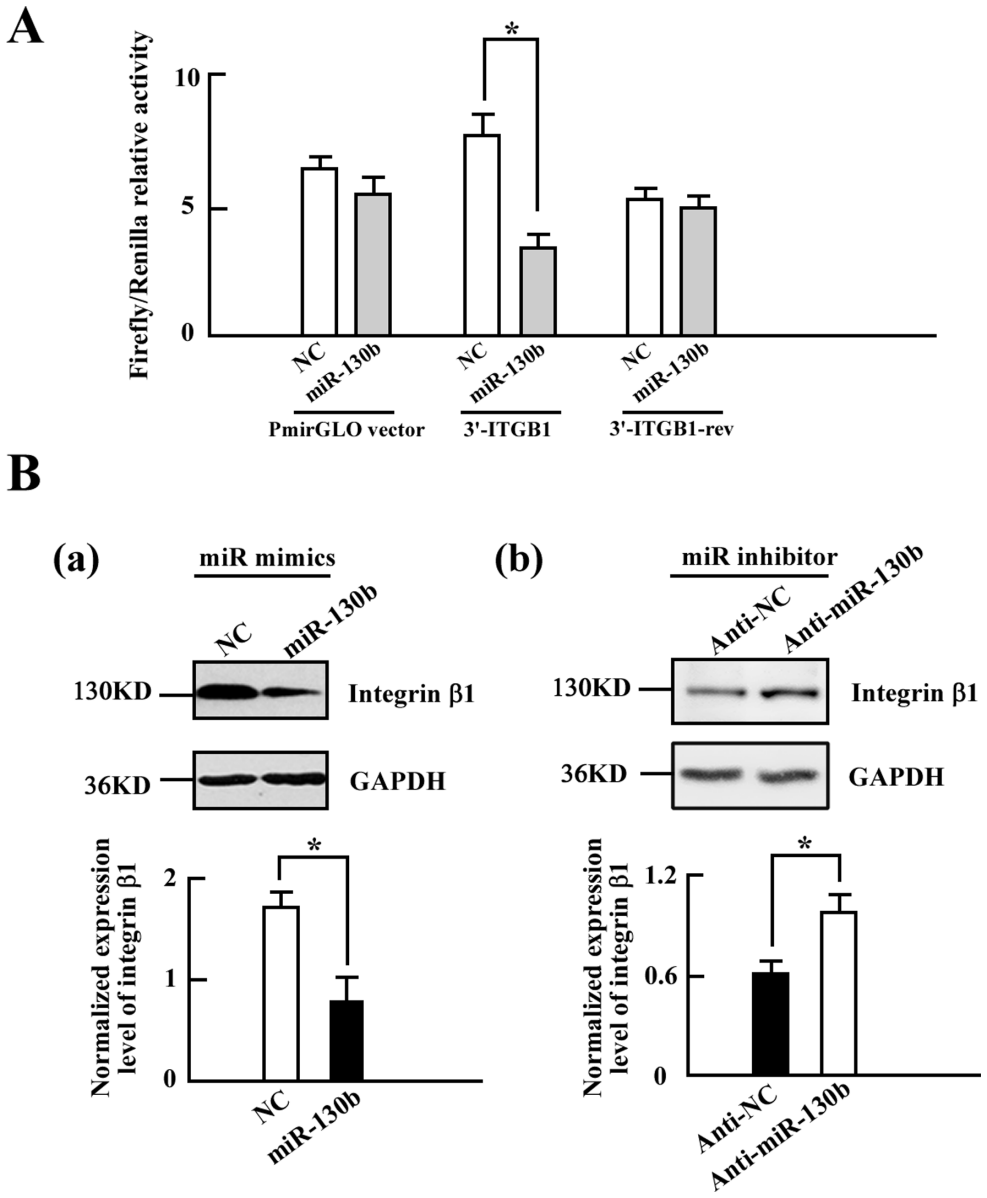
MiR-130b targets the 3′-UTR of integrin β1 to suppress its expression. *A*, The complete 3′-UTR of the integrin β1 gene (3′-ITGB1) were cloned into the pmirGlo Dual-luciferase reporter vector and co-transfected with miR-130b mimics (miR-130b) and control miR mimics (NC) into the SW480 cells, respectively. A control vector was generated by cloning the same 3′-UTR of integrin β1 gene in reverse orientation (3′-ITGB1-rev). The firefly luciferase activity was measured and normalized to Renilla luciferase activity (mean±s.d.; n = 3; *, P<0.05). *B*, Western blot analyses of integrin β1 expression in the SW480 cells transfected with NC, miR-130b mimics (*panel a*) and miR inhibitor control (Anti-NC) or miR-130b inhibitor (Anti-miR-130b) (*panel b*) from three independent experiments. Densitometry analysis of the Western blot data normalized with GAPDH (mean±s.d.; n = 3; *, *P*<0.05).

Firstly, to examine whether miR-130b was able to interact with the 3′-UTR of integrin β1, we conducted a luciferase reporter assays in the SW480 cells. The complete 3′-UTR of integrin β1 gene was cloned into the pmirGlo Dual-luciferase reporter vector. The SW-480 cells were co-transfected with pmirGlo vector containing the 3′-UTR of integrin β1 and miR-130b mimics (Fig. 4A), the result showed significantly lower expression of the luciferase compared with the cells transfected with the same reporter vector and control microRNA mimics (NC) (Fig. 4A). The effect of miR-130b on luciferase expression was eliminated when the 3′-UTR of integrin β1 was cloned in reverse orientation (3′-ITGB1-rev) (Fig. 4A). Next, to confirm the regulation of miR-130b to integrin β1 in CRC cells, we tested the integrin β1 protein level in the cells transfected with miR-130b mimics and miR-130b inhibitor (Anti-miR-130b) respectively (Fig. 4B). The results showed that the expression of integrin β1 is suppressed after transfection with miR-130b mimics (Fig. 4B (a)). Knockdown endogenous miR-130b with miR-130b inhibitor boosts integrin β1 expression (Fig. 4B (b)). Taken together, our data suggested that integrin β1 is a target gene of miR-130b.

### MiR-130b inhibits cell migration and invasion through downregulation of the expression of integrin β1

**Figure 5:**
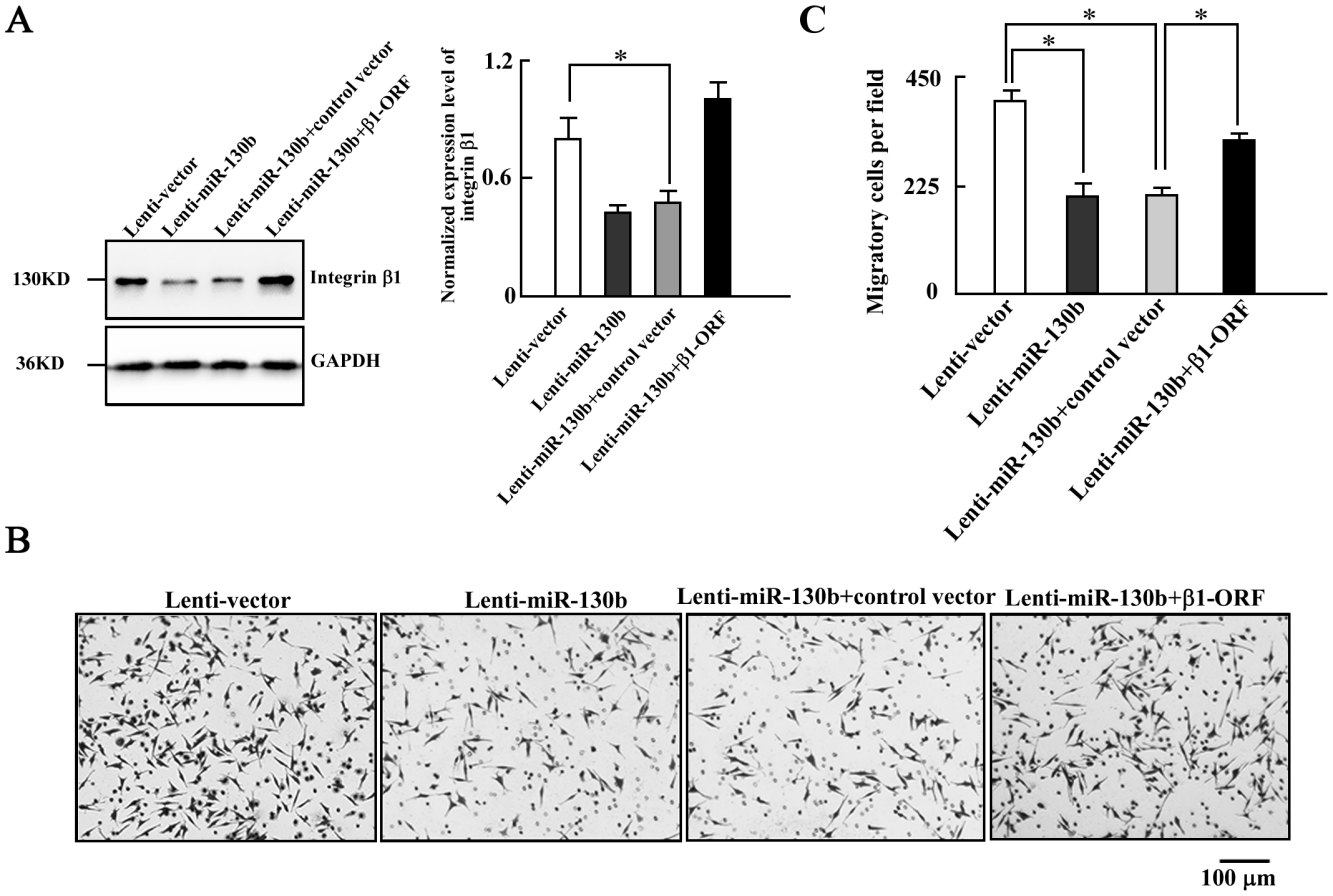
Overexpression of integrin β1-ORF rescues partly the motility of the Lenti-miR-130b cells. *A*, *Left panel*: Western blot analyses of integrin β1 expression in Lenti-control cells, Lenti-miR-130b cells, Lenti-miR-130b cells transfected with control vector or β1-ORF vector from three independent experiments. *Right panel*: Densitometry analysis of the Western blot data normalized with GAPDH (mean±s.d.; n = 3; *, *P*<0.05). *B,C*, Transwell migration assays of the cells (scale bar = 100 µm; mean±s.d.; n = 3; *, *P*<0.05). Representative images of migrated cells are shown. Lenti-miR-130b+control vector: the Lenti-miR-130b cells transfected with control vector. Lenti-miR-130b+β1-ORF: the Lenti-miR-130b cells transfected with β1-ORF vector. ORF: open reading frame.

To further investigate that suppression of integrin β1 by miR-130b results in the impaired motility of Lenti-miR-130b cells, we performed an integrin β1 rescue experiment using the Lenti-miR-130b cells. We employed an expression construct that encodes the integrin β1 open reading frame (β1-ORF) lacking the 3′-UTR [17], which yields an mRNA that is resistant to miRNA-mediated suppression. We observed a clear increase in integrin β1 expression in the Lenti-miR-130b cells transfected with β1-ORF, compared to the cells with control vector (Fig. 5A). Furthermore, cell migration assays showed that the ectopic expression of integrin β1-ORF was capable of partly recovering the motility of the Lenti-miR-130b cells by 76% (Fig. 5B and 5C).

**Figure 6:**
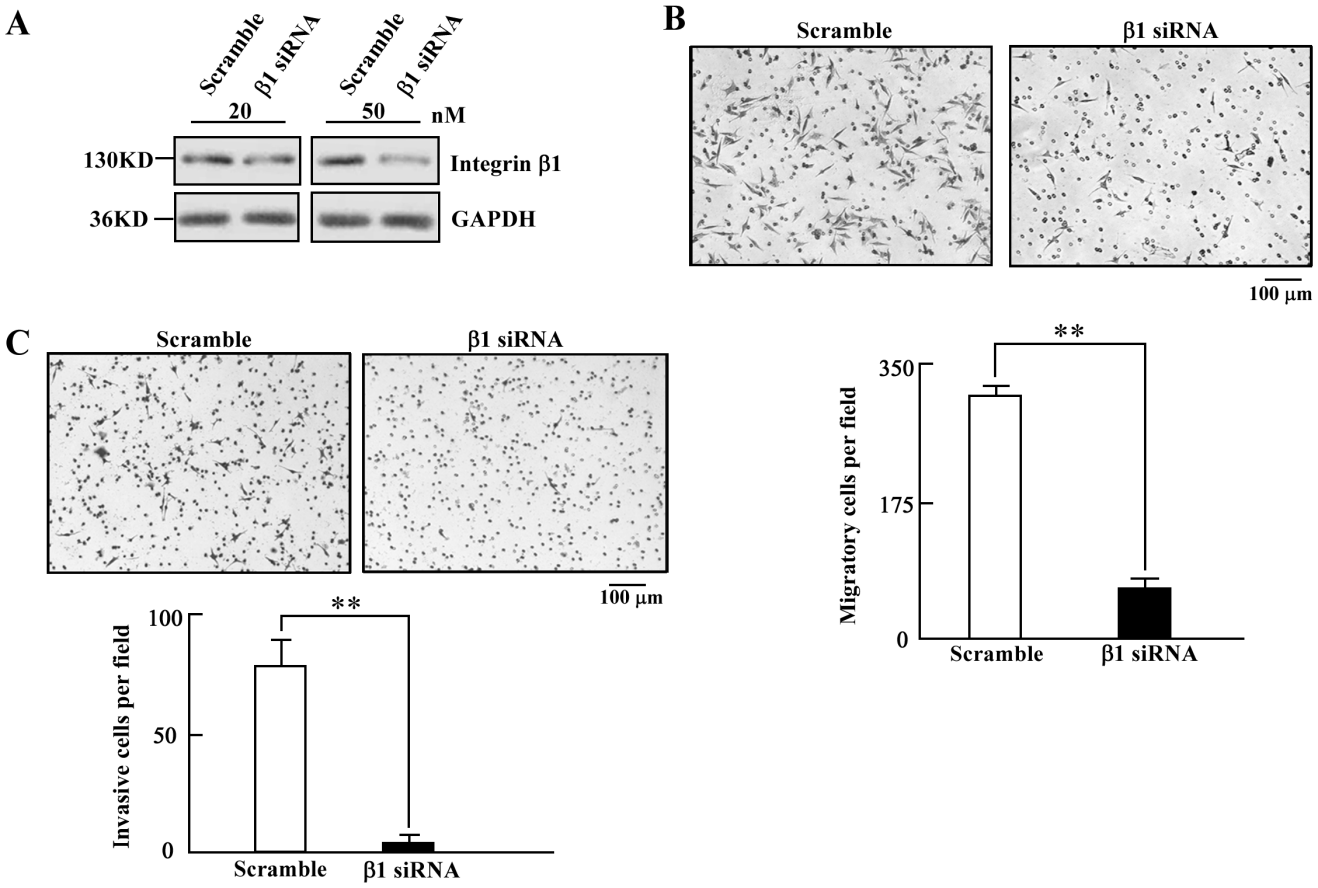
Decreased integrin β1 impairs the motility of CRC cells. *A*, Western blot analyses assessed the protein level of integrin β1 in SW480 cells transfected with a siRNA against integrin β1 (β1 siRNA) or negative control siRNA (Scramble) at 20 nM and 50 nM respectively. Each assay was independently repeated three times. *B,C*, Transwell migration (*B*) and invasion (*C*) assays of SW480 cells transfected with β1 siRNA or Scramble at 50 nM (scale bar = 100 µm; mean±s.d.; n = 3; **, *P*<0.01). Representative images of migrated cells (*B*) and invaded cells (*C*) are shown.

We subsequently inhibited integrin β1 expression using a specific siRNA [18] in the SW-480 cells (Fig. 6A). We found that cell migration (Fig. 6B) and invasion (Fig. 6C) are remarkably decreased through inhibiting integrin β1 expression with a specific siRNA. Therefore, the decrease of cell motility is achieved through the suppression of integrin β1 expression. Taken together, these findings demonstrated that miR-130b suppresses cell migration and invasion, at least in part, through downregulation of integrin β1 in CRC cells.

### Inverse correlation between miR-130b and integrin β1 expression in CRC specimens

**Figure 7:**
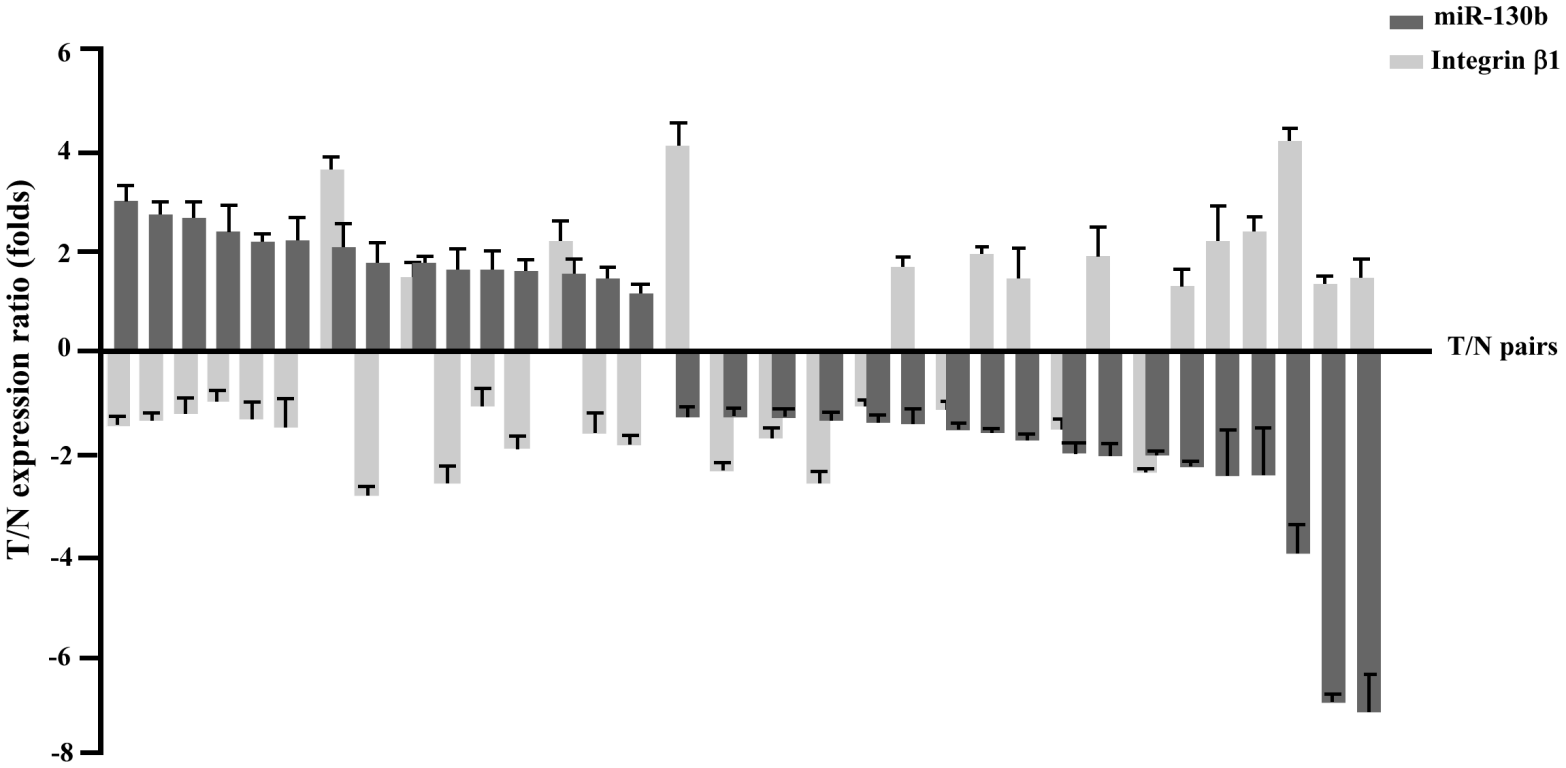
Inverse correlation between miR-130b and integrin β1 expression in human colorectal specimens. The expression of miR-130b was measured by qRT-PCR, the expression of integrin β1 was measured by Western blot analysis in a cohort of 33 matched-pairs of adjacent normal (N) and tumor (T) tissues. The relative expression ratio of miR-130b (normalized to U6) in T over N (T/N) was represented as a fold difference (columns in dark gray). The relative expression ratio of integrin β1 (normalized to GAPDH) in T over N (T/N) was represented as a fold difference (columns in light gray). mean±s.d. Each assay was independently repeated three times.

To further test the correlation between integrin β1 and miR-130b, we extended our analysis in a cohort of 33 matched-pairs of clinical adjacent normal (N) and colorectal tumor tissues (T). We analyzed the expression of miR-130b by qRT-PCR and the expression level of integrin β1 by Western blot. As shown in Fig. 7, by comparing tumors to normal tissues, an inverse correlation between miR-130b and integrin β1 expression was found in 23 of 33 (69.7%) pairs of clinical samples. Of the 23 pairs, 12 pairs showed the increased miR-130b and the decreased integrin β1; 11 pairs demonstrated the decreased miR-130b and the elevated integrin β1.

## Discussion

MicroRNA 130b (miR-130b) is significantly dysregulated in many human tumor types. However, the role of miR-130b in CRC is not well understood. In this study, we investigated the microRNA expression in colorectal cancer (CRC) using microRNA microarray profiling of tumors and adjacent normal tissue samples. We identified 48 significantly differentially expressed miRNAs associated with CRC. MiR-130b is one of the upregulated miRNAs. This data was further confirmed by qRT-PCR. To test the potential roles of the increased expression of miR-130b in CRC, we performed functional assays after constructing CRC cell line with stable overexpression of miR-130b. Our data showed that miR-130b exerts a significant inhibitory effect on motility of the CRC cells (Fig. 2), but has no effects on cell proliferation and apoptosis. It has been reported that in the *TAp63* knockout mouse model, downregulation of miR-130b by the loss of TAp63 results in an increase in tumor metastasis [28]. The repression of miR-130b by a p53 mutant results in the enhancement of ZEB1-dependent EMT and cell invasion in endometrial cancer cells [29]. All these data suggested the anti-metastatic role of miR-130b. In addition, the downregulation of miR-130b confers a multidrug-resistant phenotype in ovarian cancer cells [30]. However, another report has suggested that the overexpression of miR-130b in CD133 (+) liver tumor-initiating cells increases their self-renewal capacity and chemoresistance [31]. These results suggest that miR-130b may have a dual function as a tumor suppressor or an oncogene, which depends on the cancer type and cellular context.

In this study, we identified that integrin β1 is a novel target of miR-130b. A decreased level of integrin β1 protein was observed due to overexpression of miR-130b in CRC specimens (Fig. 3D) and in CRC cells (Fig. 3A and 3B) as well. The luciferase reporter assay showed that miR-130b binds to the 3′-UTR of integrin β1 and suppresses its expression (Fig. 4). An increase in miR-130b by miR-130b mimics transfection leads to the reduced expression of integrin β1, while knockdown miR-130b with miR-130b inhibitor results in increased integrin β1 expression. Furthermore, the impaired motility of miR-130b overexpression cells is rescued partly by the expression of integrin β1 lacking the 3′-UTR (Fig. 5). In addition, the knockdown of integrin β1 also gives rise to a decrease in cell migration and invasion (Fig. 6). These data indicated that miR-130b suppresses cell migration and invasion of CRC cells, at least in part, through downregulation of integrin β1. Moreover, the inverse correlation between miR-130b expression and integrin β1 expression was found in 23 of 33 pairs (69.7%) of CRC clinical samples. The inhibition of migration and invasion of CRC cells through direct targeting of integrin β1 is consistent with the anti-metastatic role proposed for miR-130b [28], [29]. A large body of experimental evidence supported an essential role for integrin β1 during tumor induction and invasiveness [32]–[36]. An increase in integrin β1 and activation of integrin β1-coupled signaling had been implicated in the propagation of a wide variety of human cancers [37]–[42]. In addition, blocking integrin β1 binding activity has been shown to revert the transformed phenotype of human breast cancer cells [43], [44]. Nonetheless, one miRNA may on average control more than 200 target genes [45], our data do not preclude the existence of still-uncharacterized miR-130b target genes that are involved in the motility in a manner that is masked by the consequences of altering integrin β1 expression.

Our results open a possibility that miR-130b is a miRNA with potential anti-metastasis activity in CRC. Analysis of relationship between miR-130b expression and the clinicopathological features of 32 endometrial cancer patients showed that patients with higher expression of miR-130b survived longer [29]. Similarly, in pancreatic cancer, the deregulated miR-130b is correlated with worse prognosis [46]. Previous studies also have shown that miR-130b is downregulated in aggressive papillary thyroid carcinomas [47]. Additionally, the correlations between miR-130b and progression and metastasis were reported in renal cell carcinoma [48]. As mentioned before, upregulated miR-130b was found in some types of cancer, such as: gastric cancer [5], [6], cutaneous malignant melanoma [7], bladder cancer [9] and head and neck squamous cell carcinoma [8]. Moreover, miR-130b expression is likely reduced in later stages of tumor progression in endometrial cancer patients [29]. Therefore, we postulate that the increased miR-130b in CRC might indicate less metastasis. The CRC specimens in this study were obtained between 2010 and 2011, and the long-term follow-up observations of the CRC patients are being carried out. The significance and clinical relevance of miR-130b in CRC is clearly needed to further demonstrate.

In summary, our data showed that miR-130b downregulates its novel target-integrin β1, leading to the impaired cell motility of CRC cells.

## Supporting Information

Table S1 **In microarray readouts, the 17 significantly downregulted miRNAs are indicated in human colorectal cancer specimens compared with matched non-tumor tissue.** (SAM analysis; q value (%) ≤5; fold change ≤0.05).
(DOCX)
