# Correction: MicroRNA-130b Suppresses Migration and Invasion of Colorectal Cancer Cells through Downregulation of Integrin β1

**DOI:** 10.1371/journal.pone.0091498

**Published:** 2014-03-18

**Authors:** The PLOS ONE Staff

The article title is incorrect. “The correct title is: MicroRNA-130b Suppresses Migration and Invasion of Colorectal Cancer Cells through Downregulation of Integrin β1.”

The correct Citation is: Zhao Y, Miao G, Li Y, Isaji T, Gu J, et al. (2014) MicroRNA-130b Suppresses Migration and Invasion of Colorectal Cancer Cells through Downregulation of Integrin β1. PLoS ONE 9(2): e87938. doi:10.1371/journal.pone.0087938
