# Supplementary material for: Microrna 130b Suppresses Migration and Invasion of Colorectal Cancer Cells through Downregulation of Integrin β1
Source: PLoS One. 2014 Feb 3;9(2):e87938. doi: 10.1371/journal.pone.0087938 (PMC3912181; doi:10.1371/journal.pone.0087938)
Supplement: Table S1 — In microarray readouts, the 17 significantly downregulted miRNAs are indicated in human colorectal cancer specimens compared with matched non-tumor tissue. (SAM analysis; q value (%) ≤5; fold change ≤0.05). (DOCX) [file pone.0087938.s001.docx]

**Table S1. In microarray readouts, the 17 significantly downregulted miRNAs are indicated in human colorectal cancer specimens compared with matched non-tumor tissue. (SAM analysis; q value (%) ≤5 ; fold change ≤0.05)**

| **ProbeSet Name** | **Score(d)** | **Fold Change** | **q-value(%)** | **P1-T** | **P2-T** | **P3-T** | **P1-N** | **P2-N** | **P3-N** |
| --- | --- | --- | --- | --- | --- | --- | --- | --- | --- |
| hsa-let-7e | -0.92 | 0.495 | 1.9555 | 885.58 | 313.54 | 583.61 | 1298.4 | 1087 | 1134.5 |
| hsa-miR-422a | -1.105 | 0.4883 | 0.5535 | 120.06 | 108.25 | 128.85 | 212.72 | 247.34 | 278.38 |
| hsa-miR-487b | -0.975 | 0.4784 | 0.9436 | 60.702 | 78.866 | 83.39 | 209.08 | 128.11 | 157.58 |
| hsa-miR-1293 | -1.058 | 0.4784 | 0.9436 | 28.793 | 30.643 | 31.998 | 78.509 | 51.819 | 67.089 |
| hsa-miR-215 | -1.171 | 0.4771 | 0.3194 | 39.93 | 31.615 | 76.332 | 94.26 | 62.856 | 151.28 |
| hsa-miR-100 | -0.952 | 0.4692 | 1.3612 | 171.1 | 73.525 | 122.03 | 238.62 | 308.08 | 270 |
| hsa-miR-454* | -1.139 | 0.4518 | 0.3194 | 23.704 | 17.138 | 21.587 | 61.884 | 44.093 | 36.987 |
| hsa-miR-139-5p | -1.159 | 0.4429 | 0.3194 | 33.635 | 20.798 | 31.465 | 60.978 | 61.873 | 71.356 |
| hsa-miR-374a* | -1.108 | 0.4054 | 0.5535 | 48.431 | 67.917 | 60.674 | 246.23 | 138.54 | 114.62 |
| hsa-miR-497 | -1.476 | 0.3891 | 0 | 77.716 | 43.745 | 57.755 | 180.86 | 127.42 | 146.5 |
| hsa-miR-132* | -1.339 | 0.3863 | 0 | 104.33 | 113.62 | 108.07 | 371.33 | 260.77 | 244.34 |
| hsa-miR-181c* | -1.341 | 0.3739 | 0 | 79.925 | 81.425 | 81.062 | 313.44 | 178.1 | 197.97 |
| hsa-miR-585 | -1.266 | 0.3612 | 0 | 19.351 | 24.338 | 23.059 | 101.42 | 57.363 | 49.211 |
| hsa-miR-30a | -1.418 | 0.3529 | 0 | 83.459 | 81.577 | 98.531 | 346.3 | 212.93 | 226.73 |
| hsa-miR-150 | -1.557 | 0.3524 | 0 | 301.28 | 156.03 | 180.6 | 740.18 | 433.33 | 622.32 |
| hsa-miR-181a* | -1.48 | 0.3474 | 0 | 61.324 | 66.065 | 74.754 | 243.2 | 176.33 | 179.92 |
| hsa-miR-195 | -1.912 | 0.2626 | 0 | 270.6 | 187.64 | 262.53 | 896.03 | 942.12 | 915.71 |
